# Supplementary material for: Elp2 mutations perturb the epitranscriptome and lead to a complex neurodevelopmental phenotype
Source: Nat Commun. 2021 May 11;12:2678. doi: 10.1038/s41467-021-22888-5 (PMC8113450; doi:10.1038/s41467-021-22888-5)
Supplement: Supplementary file 1 — Supplementary Information [file 41467_2021_22888_MOESM1_ESM.pdf]

Supplementary Information

Elp2 mutations perturb the epitranscriptome and lead to a complex neurodevelopmental phenotype

Kojic *et al*.

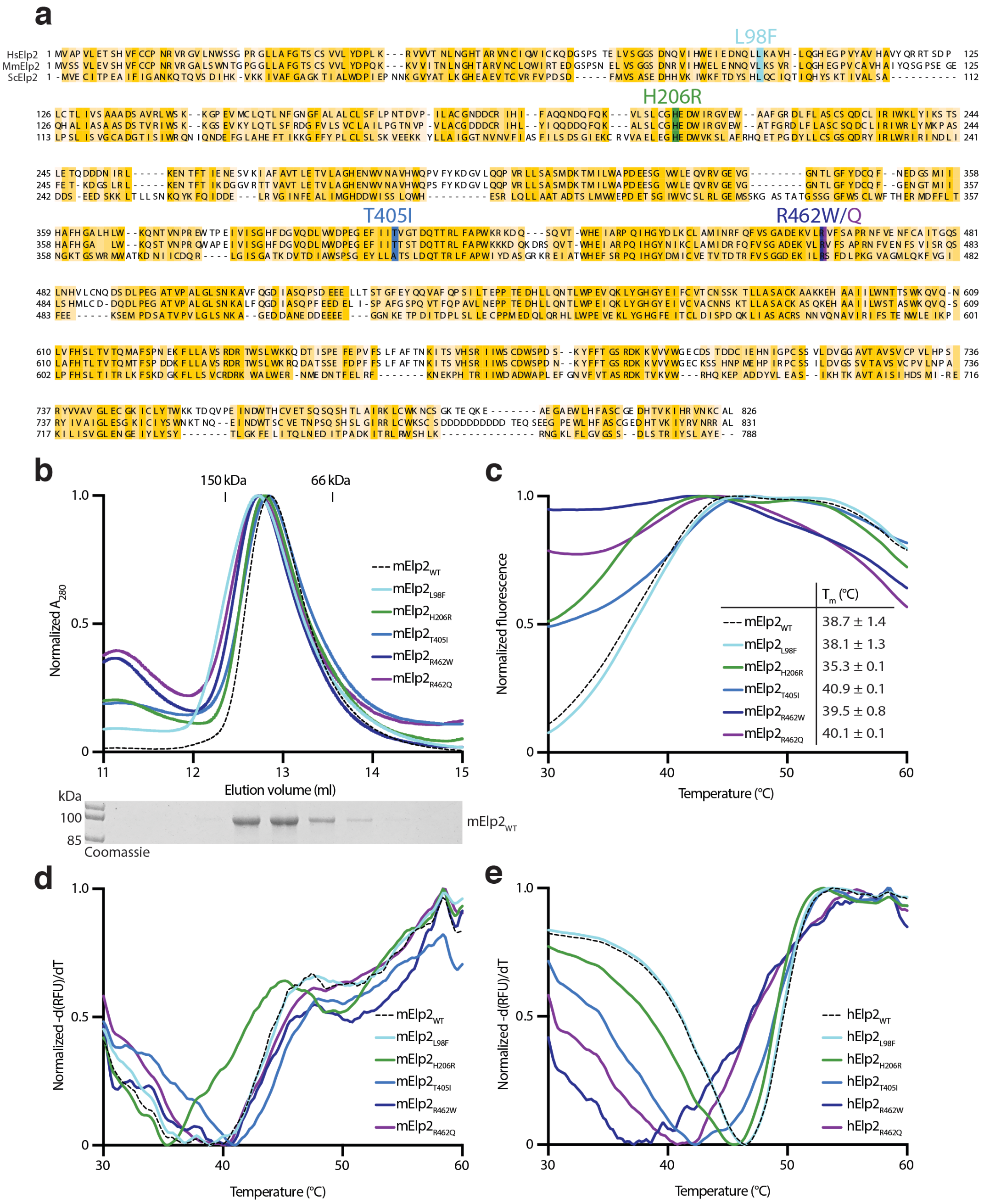

Supplementary Figure 1. Mutations found in patients with intellectual disability and autism impair the mElp2 protein stability. (**a**) Alignment of *Homo sapiens* (HsElp2), *Mus musculus* (MmElp2), *Saccharomyces cerevisiae* (ScElp2) Elp2 protein sequences with highlighted location of patient-derived mutations. Residues are labelled in orange depending on the conservation score. (**b**) Purification of mouse Elp2 wild-type ($mElp2_{WT}$) and mutant proteins ($mElp2_{L98F}$, $mElp2_{H206R}$, $mElp2_{T405I}$, $mElp2_{R462W}$, $mElp2_{R462Q}$). Gel filtration profiles from S200 Increase 10/300 GL column for mElp2 variants (top) and Coomassie-stained SDS-PAGE gel for $mElp2_{WT}$ (bottom). $n = 3$ independent experiments. (**c**) Averaged melting curves from thermal shift assay for mElp2 variants with calculated melting temperatures (T_m) (mean ± SD). (**d**) Normalized first derivative plot from thermal shift assay experiments for mElp2 variants. (**e**) Normalized first derivative plot from thermal shift assay experiments for hElp2 variants. $n = 3$ independent measurements for (**c**-**e**). Source data are provided as a Source Data file.

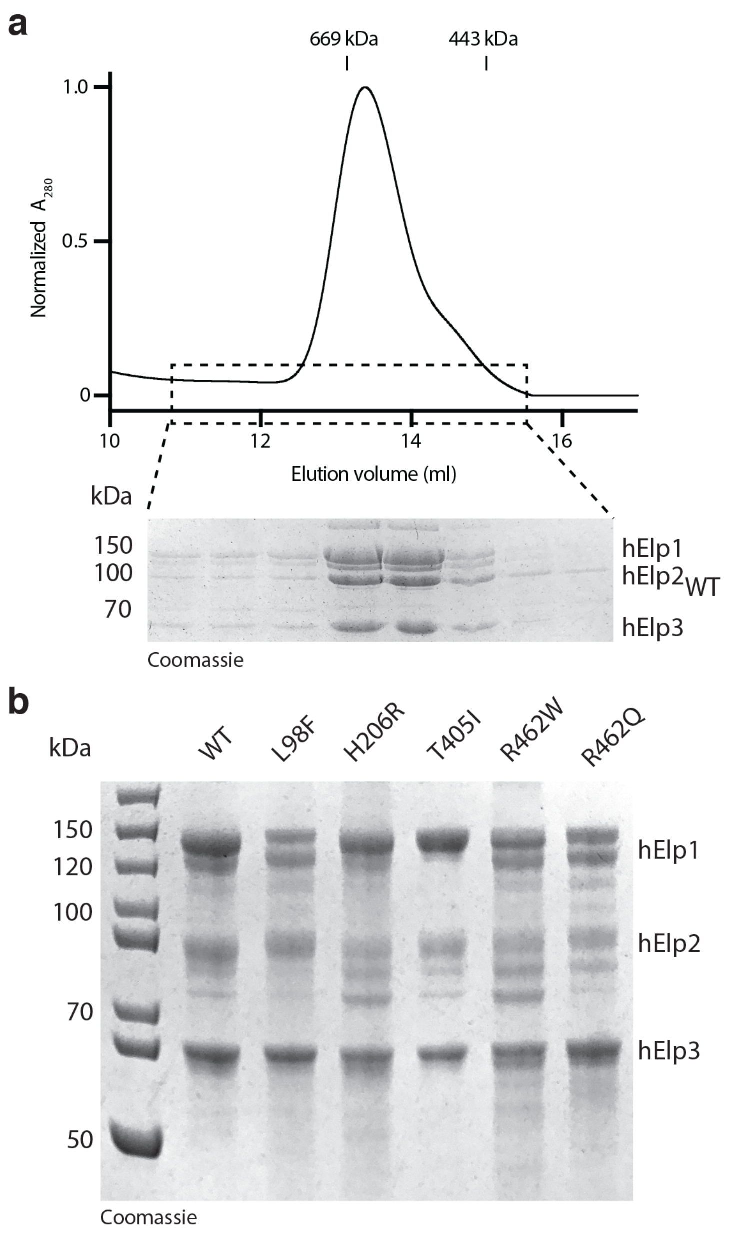

Supplementary Figure 2. Purified hElp123 subcomplex variants. (**a**) Gel filtration profile from Superose 6 Increase 10/300 GL column for hElp123 (top) and SDS PAGE of the respective fractions stained with Coomassie (bottom). (**b**) Coomassie-stained SDS-PAGE gel of purified human Elp123 wild-type ($hElp123_{WT}$) and mutant variants ($hElp2_{L98F}3$, $hElp2_{H206R}3$, $hElp2_{T405I}3$, $hElp2_{R462W}3$, $hElp2_{R462Q}3$) with hElp123 subunits marked on the right side of the gel. n = 3 independent experiments for both (**a**) and (**b**). Source data are provided as a Source Data file.

a

Genotype	9-10 dpc Expected	9-10 dpc Observed	14-15 dpc Expected	14-15 dpc Observed	16-17 dpc Expected	16-17 dpc Observed	18-18.5 dpc Expected	18-18.5 dpc Observed	Weaned Expected	Weaned Observed
Elp2 $^{+/+}$	10.5	17	9	11	6.25	9	Not analyzed		14.25	19
Elp2 $^{H206R/+}$	10.5	10	9	7	6.25	10			14.25	16
Elp2 $^{+/-}$	10.5	9	9	13	6.25	6			14.25	22
Elp2 $^{H206R/-}$	10.5	6	9	5	6.25	0			14.25	0
Total	42		36		25				57	
Elp2 $^{+/+}$	11.75	15	8	7	Not analyzed		Not analyzed		15.25	18
Elp2 $^{R464W/+}$	11.75	9	8	12					15.25	28
Elp2 $^{+/-}$	11.75	17	8	13					15.25	15
Elp2 $^{R464W/-}$	11.75	6	8	0					15.25	0
Total	47		32						61	
Elp2 $^{+/+}$	9.5	7	8.25	7	10.5	14	6.5	10	21	23
Elp2 $^{H206R/+}$	9.5	11	8.25	9	10.5	9	6.5	5	21	32
Elp2 $^{R464W/+}$	9.5	12	8.25	6	10.5	13	6.5	8	21	29
Elp2 $^{H206R/R464W}$	9.5	8	8.25	11	10.5	6	6.5	3	21	0
Total	38		33		42		26		84	
Elp2 $^{+/+}$	11.5	23	9.5	10	Not analyzed		Not analyzed		18	29
Elp2 $^{R464W/+}$	23	29	19	28					36	43
Elp2 $^{R464W/R464W}$	11.5	7	9.5	0					18	0
Total	46		38						72	

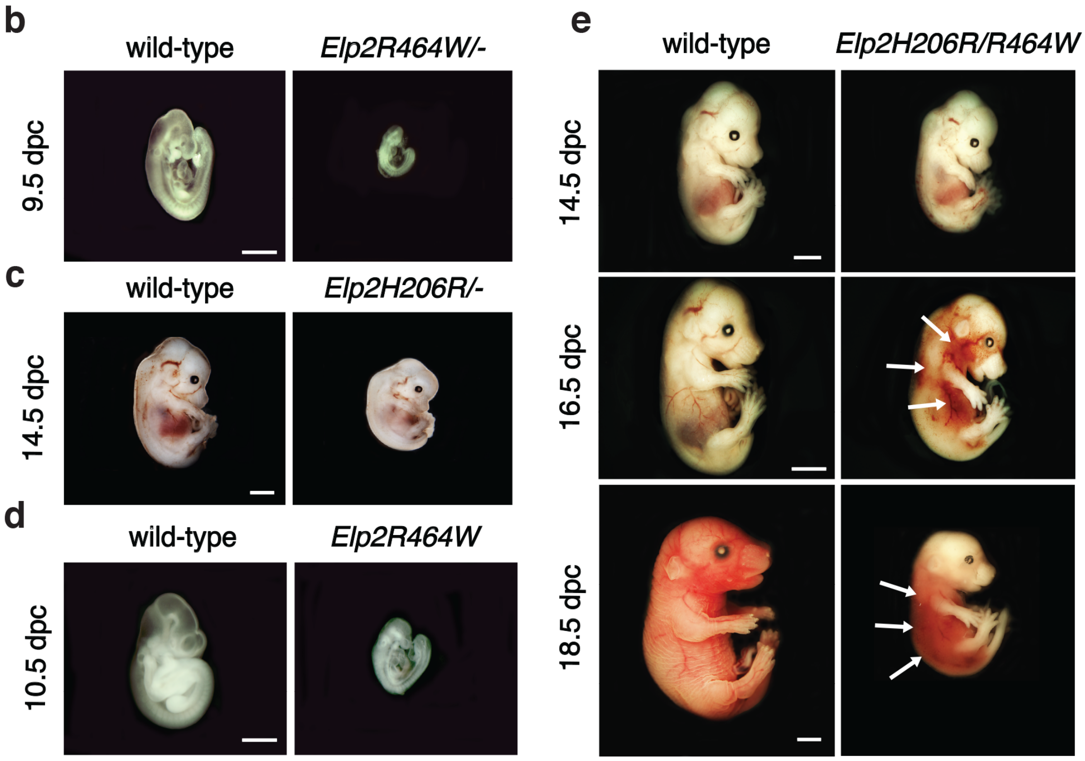

Supplementary Figure 3. Embryonic lethality of *Elp2* mutations on C57BL/6 genetic background. (**a**) Expected and observed genotypes based on Mendelian frequency from *Elp2*$^{H206R/+}$ and *Elp2*$^{+/-}$ (first row), *Elp2*$^{R464W/+}$ and *Elp2*$^{+/-}$ (second row), *Elp2*$^{H206R/+}$ and *Elp2*$^{R464W/+}$ (third row), and *Elp2*$^{R464W/+}$ (last row) intercrosses at different stages of development. Representative whole-mount images are shown for: (**b**) compound heterozygous embryos for *Elp2R464W* and *Elp2* null mutation (*Elp2R464W*/-), (**c**) compound heterozygotes for *Elp2H206R* and *Elp2* null mutation (*Elp2H206R*/-), (**d**) *Elp2R464W* homozygous (*Elp2R464W*) and (**e**) compound heterozygous embryos for *Elp2R464W* and *Elp2H206R* mutations (*Elp2H206R*/*R464W*), and matching littermate controls for each genotype at indicated stages of the embryonic development. Arrows point to haemorrhaging in the mutant embryos. Number of embryos per genotype is indicated in (**a**); representative images are shown (**b**-**e**). Scale bars, 1 mm. Abbreviations: dpc - days post-coitum.

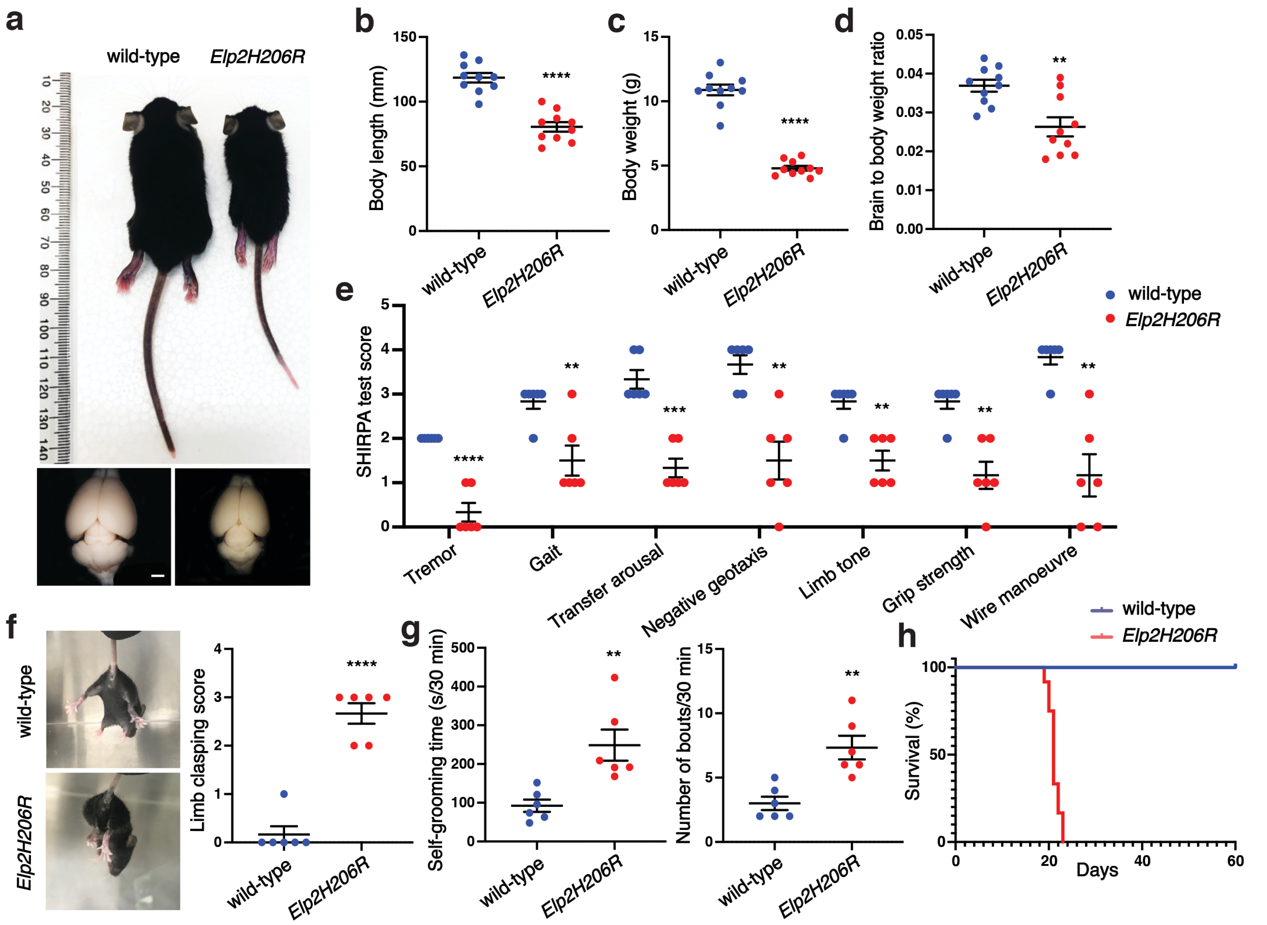

Supplementary Figure 4. ***Elp2H206R* mice show signs of developmental delay, microcephaly, motor and autism-like defects.** (**a**) Appearance of an *Elp2H206R* homozygous mouse on C57BL/6 genetic background and its control littermate (upper panel), as well as their dissected brains (lower panel; scale bar, 2 mm) at postnatal day 21 (P21). (**b**) Body length, (**c**) weight and (**d**) relative brain weight of the indicated genotypes were quantified at P21. (**e**) Significant effects of *Elp2H206R* on scores for SHIRPA test at P21. (**f**) Representative image (left) and scoring (right) of hindlimb clasping in P21 wild-type and mutant mice. (**g**) Total self-grooming time and number of self-grooming bouts in *Elp2H206R* mutants vs. control at P21 (recorded for 30 min in a home cage). For (**a-d**) $n = 10$ (5 males and 5 females) and for (**e-g**) $n = 6$ (3 males and 3 females). (**h**) Kaplan–Meier curve of mouse survival ($n = 12$). Statistical analysis: unpaired two-tailed t-test ($\alpha = 0.05$) with Welch's correction. Holm-Sidak correction was applied to adjust for multiple comparisons (**e**). Statistically significant differences are indicated (**$p \leq 0.01$; ***$p \leq 0.001$; ****$p \leq 0.0001$). Data represent mean ± SEM. Source data are provided as a Source Data file.

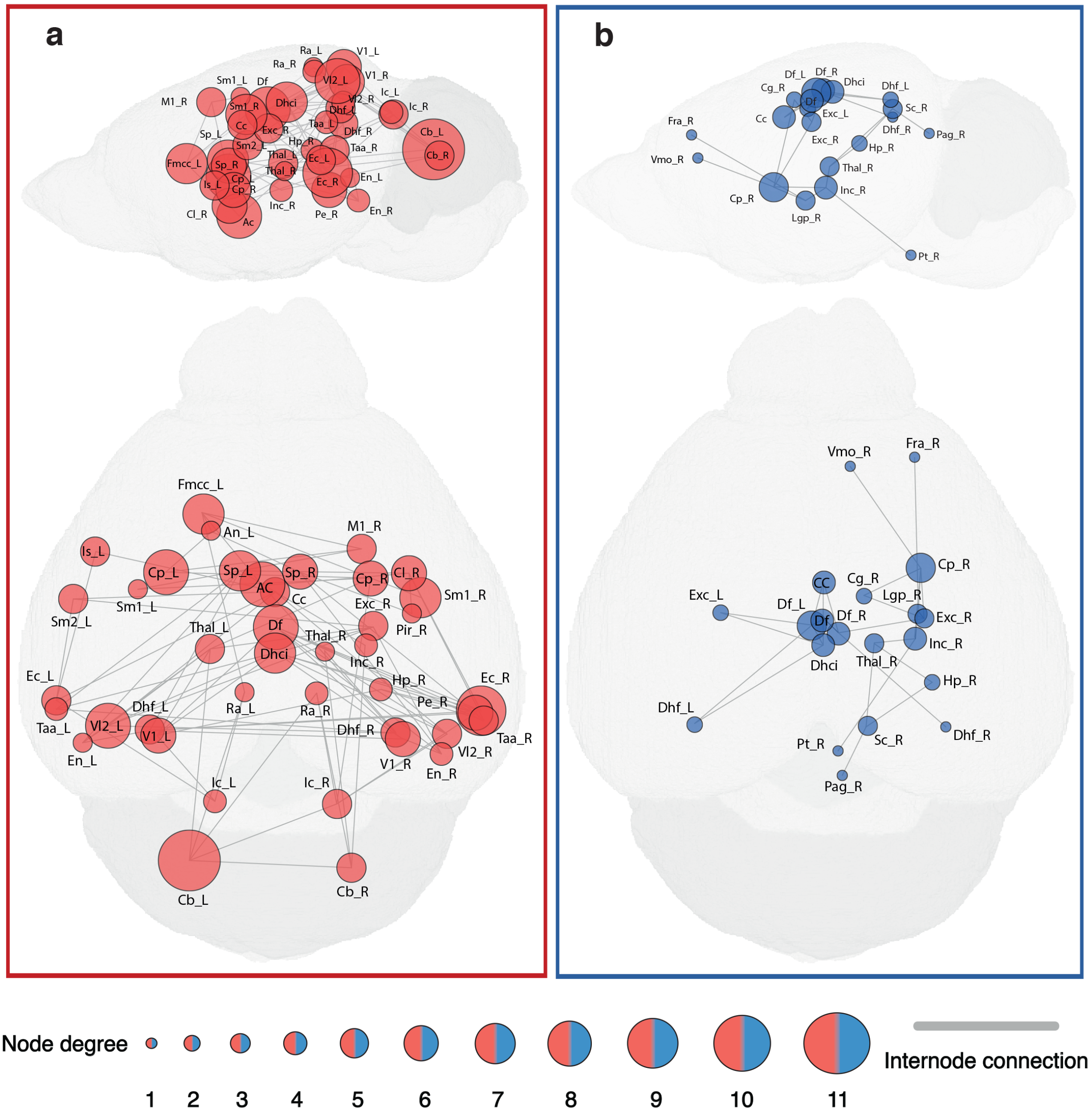

Supplementary Figure 5. Altered brain connectivity in *Elp2H206R* mice. Structural connectome maps generated via whole brain white matter tractography showing (**a**) increased and (**b**) decreased connectivity in *Elp2H206R* homozygous mice in comparison to their wild-type littermates *(n* = 6 animals per genotype) at postnatal day 21-23. Specific structures (nodes) within the brain are represented as circles, scaled according to the number of abnormal connections associated with that node (node degree). The maps are presented in sagittal (upper panels) and axial (lower panels) planes. Nodes exhibiting decreased connectivity in the brains of *Elp2H206R* mice are depicted in blue, while nodes with increased connectivity are shown in red. Nodes with a degree higher than 2 are included in the increased connectivity network for visualization purposes. Abbreviations: Ac - anterior commissure, An - accumbens nucleus, Cb - cerebellum, Cc - corpus callosum, Cc/Exc - corpus callosum/external capsule, Cg - cingulum, Cl - claustrum, Cp - caudate putamen, Df - dorsal fornix, Dhci - dorsal hippocampal commissure inclusion, Dhf - dorsal hippocampal fissure, Ec - ectorhinal cortex, En - entorhinal cortex, Fmcc - forceps minor of the corpus callosum, Fra - frontal association cortex, Hp - hippocampus, Ic - inferior colliculus, Inc - internal capsule, Is - insular cortex, L – left, Lgp - lateral globus pallidus, M1 - primary motor cortex, Pag - periaqueductal grey, Pe - perirhinal cortex, Pir - piriform nucleus, Pt - pyramidal tract, R – right, Ra - retrosplenial area, Sc - superior colliculus, Sm1 - primary somatosensory cortex, Sm2 - secondary somatosensory cortex, Sp - septum, Taa - temporal association area, Thal - thalamus, V1 - primary visual cortex, Vl2 – secondary visual cortex (lateral), Vmo - ventromedial orbital cortex.

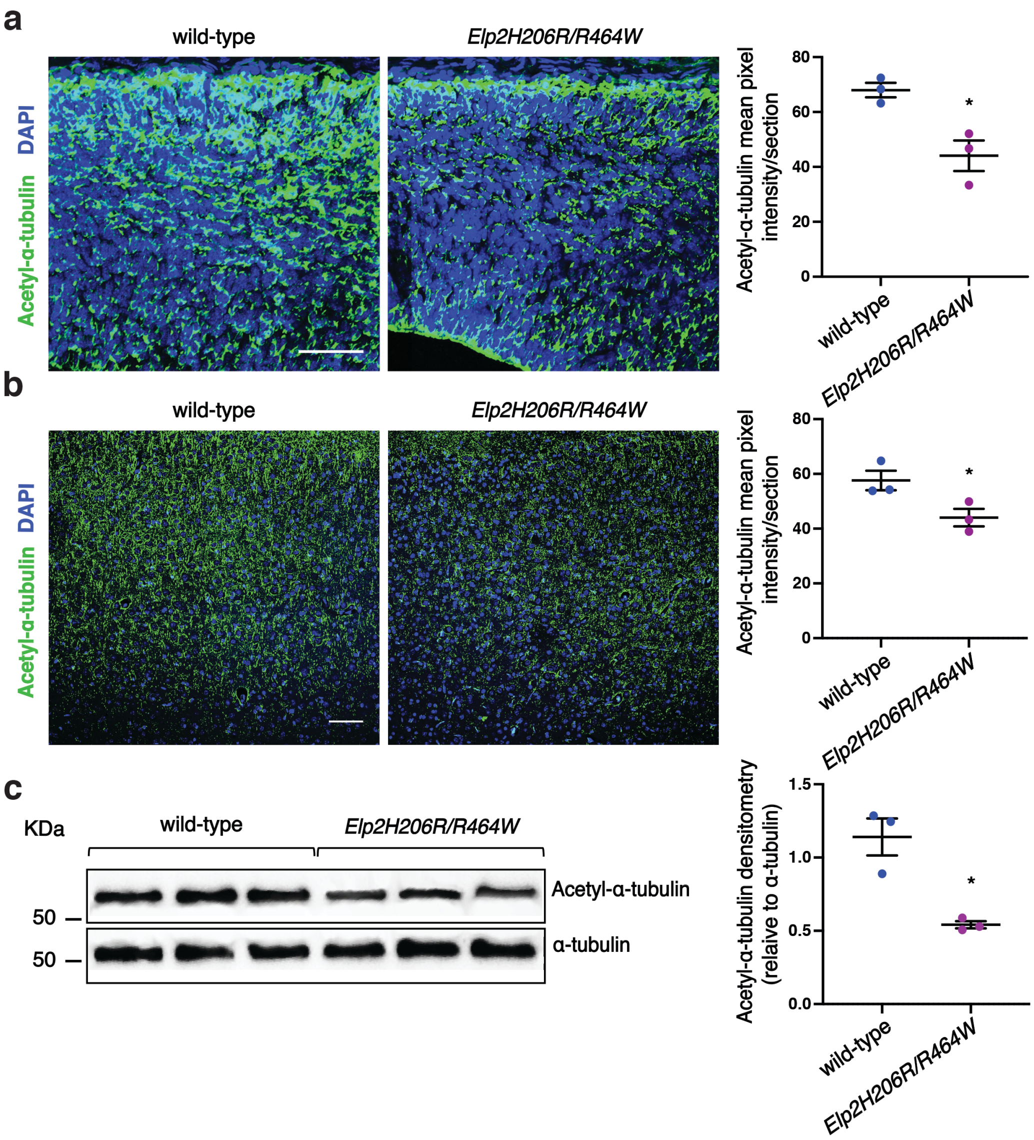

Supplementary Figure 6. Acetylation defects in the brain cortex of *Elp2* mutant mice. (**a**) Acetyl-α-tubulin immunostaining and mean pixel intensity quantification on coronal brain sections of 14.5 days post-coitum and (**b**) adult (2-months-old) wild-type and *Elp2H206R/R464W* mice. Sections were counterstained with DAPI. (**c**) Quantification of acetyl-α-tubulin expression relative to α-tubulin in adult brains by western blot. $n = 3$ animals per genotype and 3 sections per animal (**a** and **b**); representative images are shown. Scale bars, 100 µm. Statistical analysis: unpaired two-tailed *t*-test ($\alpha = 0.05$) with Welch's correction. Statistically significant differences are indicated (*$p \leq 0.05$: for (**a**) $p = 0.0329$; for (**b**) $p = 0.0483$; for (**c**) $p = 0.0373$). Data represent mean ± SEM. Source data are provided as a Source Data file.

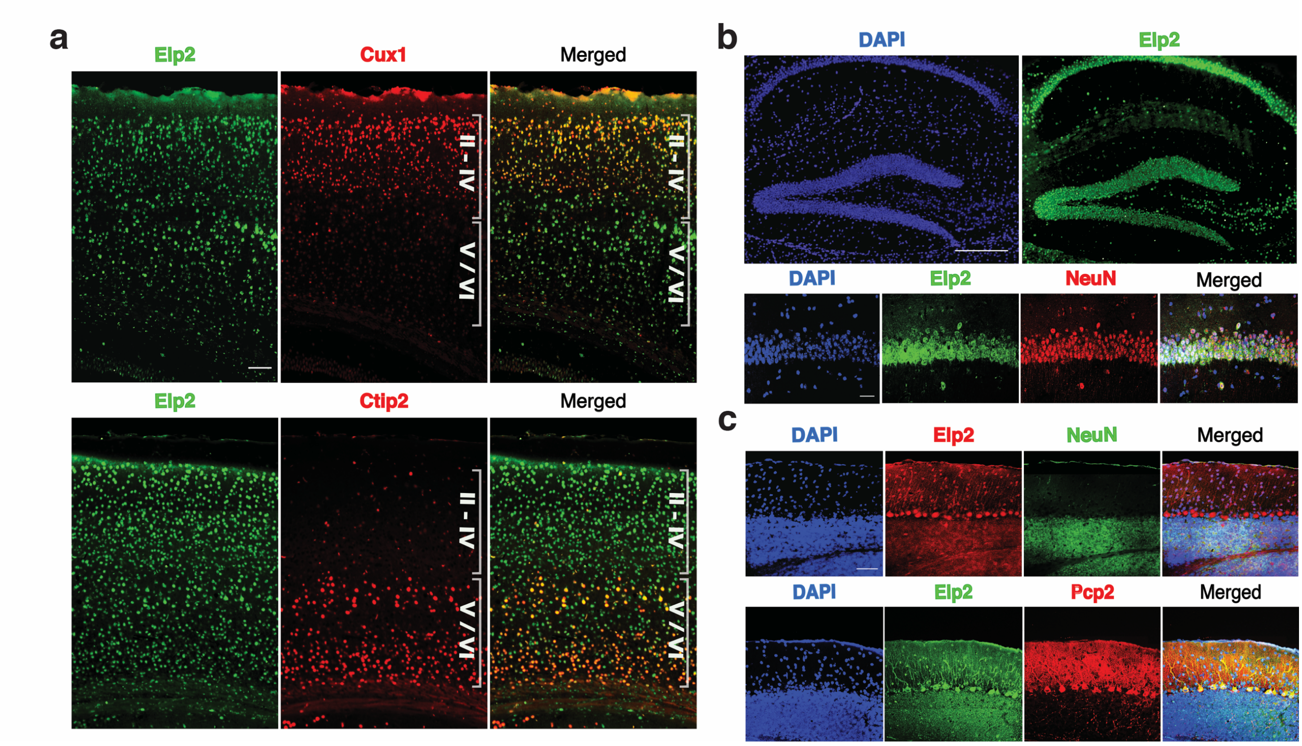

Supplementary Figure 7. Elp2 is expressed by cortical, hippocampal and cerebellar neurons. Immunolabelling of brain sections of adult (2-months-old) wild-type mice showing Elp2 expression in (**a**) upper (II-IV; Cux1-labelled neurons) and lower (V/VI; Ctip2-labelled neurons) cortical layers, (**b**) hippocampal neurons (marked by NeuN) and (**c**) granule (NeuN-labelled) and Purkinje neurons (Pcp2-labelled) in the cerebellum. Sections were counterstained with DAPI. $n = 3$ animals and 3 sections per animal; representative images are shown. Scale bars, 100 µm (**a**), 200 µm (**b** upper panels) and 50 µm (**b** lower panels and **c**).

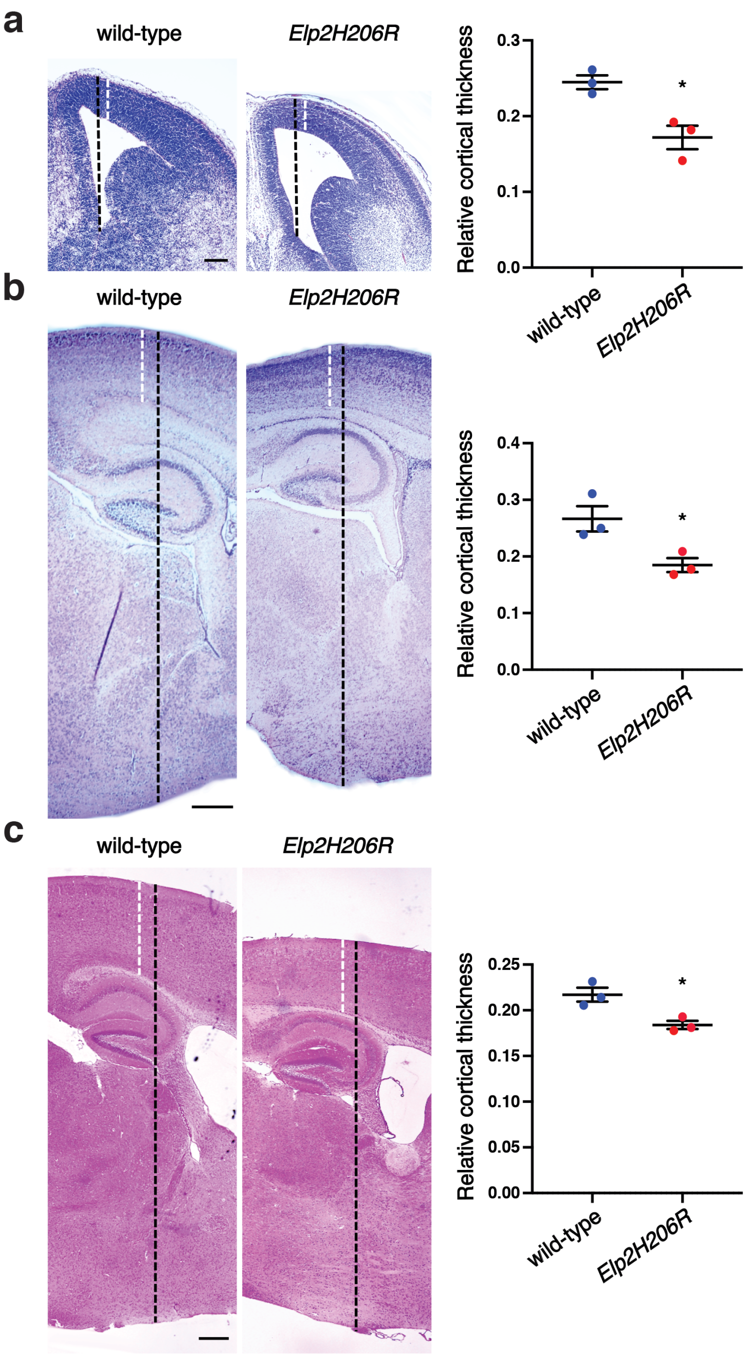

Supplementary Figure 8. Reduced cortical thickness in *Elp2H206R* mice. (**a**) H&E-stained brain sections of 14.5 days post-coitum embryos (coronal sections), (**b**) 7-days-old and (**c**) adult (2-months-old; sagittal sections) *Elp2H206R* and wild-type mice. Thicknesses of the cortical layers (white lines) were quantified relative to the section thicknesses indicated by black lines. $n = 3$ animals per genotype and 3 sections per animal; representative images are shown. Scale bars, 100 µm (**a**) and 500 µm (**b** and **c**). Statistical analysis: unpaired two-tailed t-test ($\alpha = 0.05$) with Welch's correction. Statistically significant differences are indicated (*$p \leq 0.05$: for (**a**) $p = 0.0234$; for (**b**) $p = 0.0471$: for (**c**) $p = 0.0288$). Data represent mean ± SEM. Source data are provided as a Source Data file.

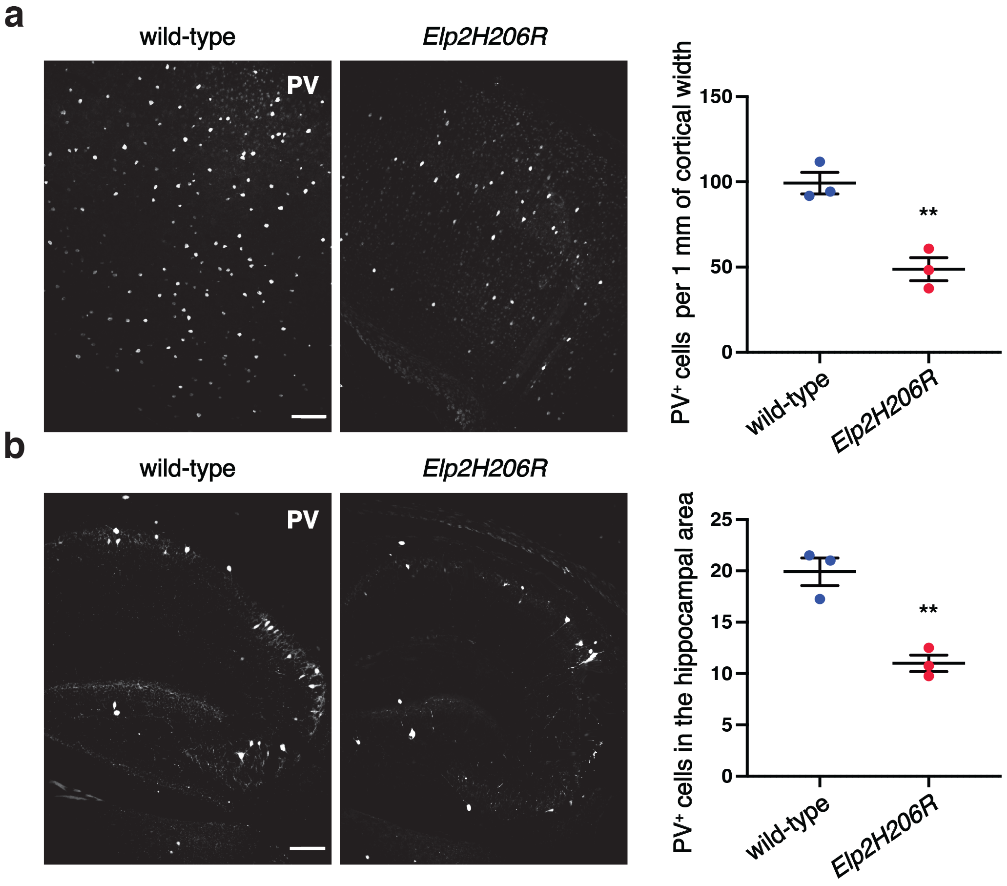

Supplementary Figure 9. Interneuron defects in *Elp2H206R* mice. (**a**) Immunofluorescence labelling and quantification of cortical and (**b**) hippocampal parvalbumin (PV)-expressing interneurons in adult (2-months-old) brains of *Elp2H206R* mice and their wild-type littermates. n = 3 animals per genotype and 4 sections per animal; representative images are shown. Scale bars, 100 µm. Statistical analysis: unpaired two-tailed t-test ($\alpha = 0.05$) with Welch's correction. Statistically significant differences are indicated (**$p \leq 0.01$: for (**a**) $p = 0.0055$: for (**b**) $p = 0.0084$). Data represent mean ± SEM. Source data are provided as a Source Data file.

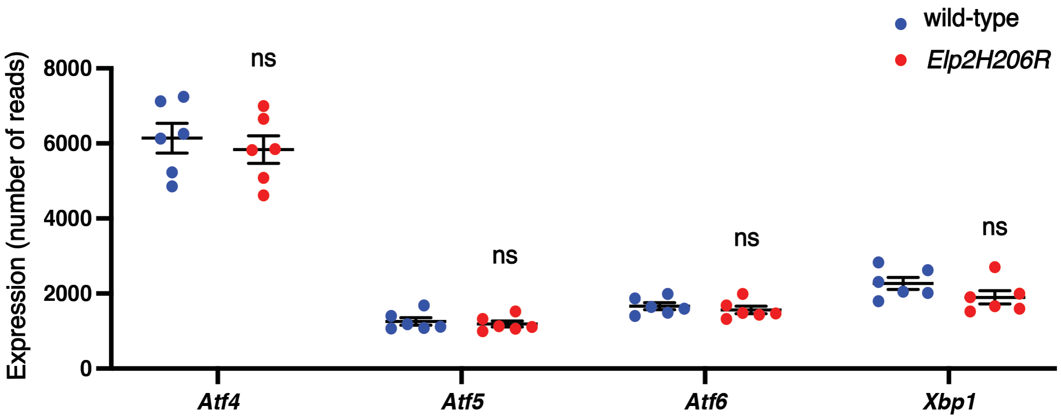

Supplementary Figure 10. Expression of unfolded protein response genes in the developing brain of *Elp2H206R* mice. Transcriptomic analysis of the key genes involved in the unfolded protein response, namely *Atf4* and *Atf5* (PERK pathway), *Atf6* and *Xbp1* (IRE-1α pathway) in the forebrain extracts of *Elp2H206R* vs. wild-type 14.5 days post-coitum embryos (n = 6 animals per genotype). Statistical analysis: unpaired two-tailed t-test (α = 0.05). Holm-Sidak correction was applied to adjust for multiple comparisons. Statistically significant differences are indicated (ns - not significant: p = 0.7053 for *Atf4*; p = 0.9346 for both *Atf5* and *Atf6*; p = 0.6539 for *Xbp1*). Source data are provided as a Source Data file.

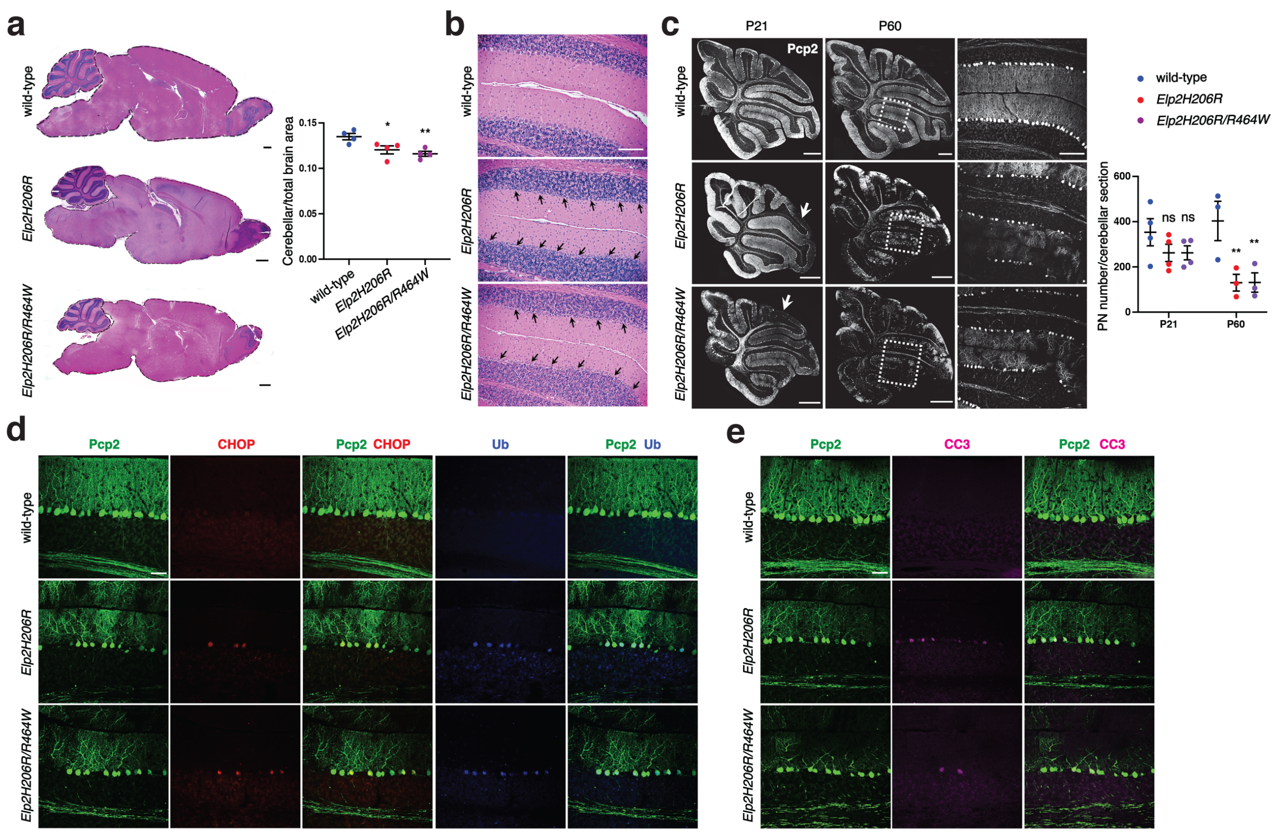

Supplementary Figure 11. Protein misfolding and ER-stress-induced apoptosis in Purkinje neurons in *Elp2* mutant mice. (**a**) H&E-stained sagittal brain sections of wild-type, *Elp2H206R* homozygous and *Elp2H206R/R464W* compound heterozygous mice at postnatal day 60 (P60). Cerebellar area relative to the whole-brain area was quantified (marked by dashed line). (**b**) Magnified cerebellar lobules from (**a**). Arrows indicate Purkinje neuron (PN) loss. (**c**) Pcp2 immunofluorescence and PN quantification in *Elp2* mutant and control mice at P21 and P60. Arrows indicate PN loss and white rectangles represent magnified areas. (**d**) Immunofluorescence staining of P60 cerebellar sections for expression of CHOP, ubiquitin (Ub) and (**e**) cleaved caspase-3 (CC3) in Purkinje neurons (Pcp2). For (**a**), (**b**) and (**c**) at P21 $n = 4$ mice per genotype, for (**c**) at P60, (**d)** and (**e**) $n = 3$ animals per genotype and 3 sections per animal; representative images are shown. Scale bars, 500 µm (**a** and **c** left and middle panels), 100 µm (**b** and **c** right panels) and 50 µm (**d** and **e**). Statistical analysis: one-way ANOVA ($\alpha = 0.05$) (**a**) and two-way ANOVA ($\alpha = 0.05$) (**c**) with a Dunnett's post-hoc test. Statistically significant differences are indicated (*$p \leq 0.05$: $p = 0.0356$; **$p \leq 0.01$: for (**a**) $p = 0.0094$; for (**c**) $p = 0.0061$ for *Elp2H206R* and $p = 0.0062$ for *Elp2H206R/R464W*; ns - not significant: $p = 0.3254$ for *Elp2H206R* and $p = 0.3266$ for *Elp2H206R/R464W*). Data represent mean ± SEM. Source data are provided as a Source Data file.

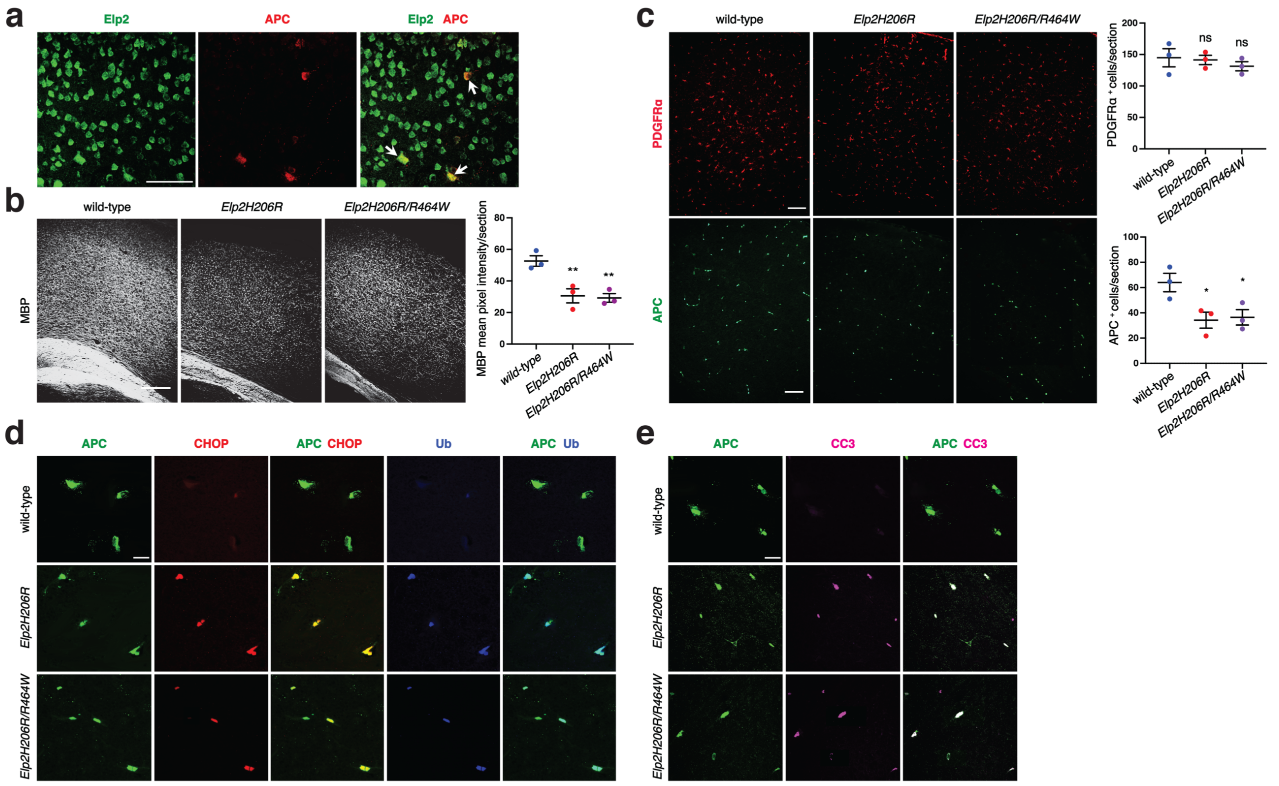

Supplementary Figure 12. Unfolded protein response-mediated myelin destruction in the brain of *Elp2* mutant mice. (**a**) Immunolabelling of brain sections of adult (2-months-old) wild-type mice showing Elp2 expression in oligodendrocytes (APC-labelled). (**b**) Myelin basic protein (MBP) immunostaining on sagittal brain sections of adult wild-type, *Elp2H206R* homozygous and *Elp2H206R/R464W* compound heterozygous mice, and mean pixel intensity quantification. (**c**) Immunostaining and quantification of oligodendrocyte progenitor cells (PDGFRα-labelled) and mature oligodendrocytes (APC) in the brain of wild-type and the *Elp2* mutant animals. (**d**) Immunofluorescence staining of cortical sections for CHOP, ubiquitin (Ub) and (**e**) cleaved caspase-3 (CC3) expression in oligodendrocytes (APC). $n = 3$ animals per genotype and 3 sections per animal; representative images are shown. Scale bars, 100 µm (**a** and **c**), 500 µm (**b**) and 20 µm (**d** and **e**). Statistical analysis: one-way ANOVA ($\alpha = 0.05$) with a Dunnett's post-hoc test. Statistically significant differences are indicated ($*p \leq 0.05$: $p = 0.033$ for *Elp2H206R* and $p = 0.0445$ for *Elp2H206R/R464W*; $**p \leq 0.01$: $p = 0.0086$ for *Elp2H206R* and $p = 0.0065$ for *Elp2H206R/R464W*; ns - not significant: $p = 0.9575$ for *Elp2H206R* and $p = 0.5774$ for *Elp2H206R/R464W*). Data represent mean ± SEM. Source data are provided as a Source Data file.

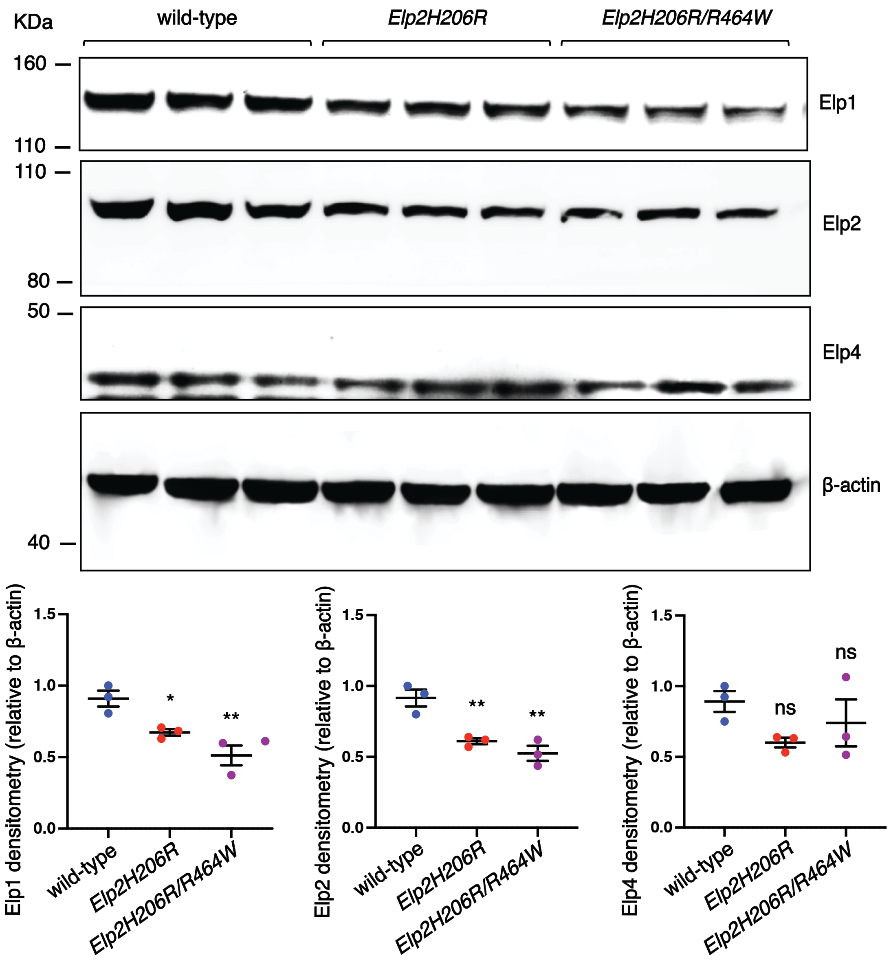

Supplementary Figure 13. Expression of Elongator subunits in the brain tissue of *Elp2* mutant mice. Western blot analysis of Elp1, Elp2 and Elp4 expression in the brain lysates of adult (2-months-old) wild-type, *Elp2H206R* and *Elp2H206R/R464W* mice. The levels of expression were quantified and normalized with β-actin. $n = 3$ animals per genotype. Statistical analysis: one-way ANOVA ($\alpha = 0.05$) with a Dunnett's post-hoc test. Statistically significant differences are indicated (*$p \leq 0.05$: $p = 0.0353$; **$p \leq 0.01$: for Elp1 densitometry $p = 0.0034$; for Elp2 densitometry $p = 0.0071$ for *Elp2H206R* and $p = 0.0021$ for *Elp2H206R/R464W*; ns - not significant: $p = 0.1724$ for *Elp2H206R* and $p = 0.5383$ for *Elp2H206R/R464W*). Data represent mean ± SEM. Source data are provided as a Source Data file.

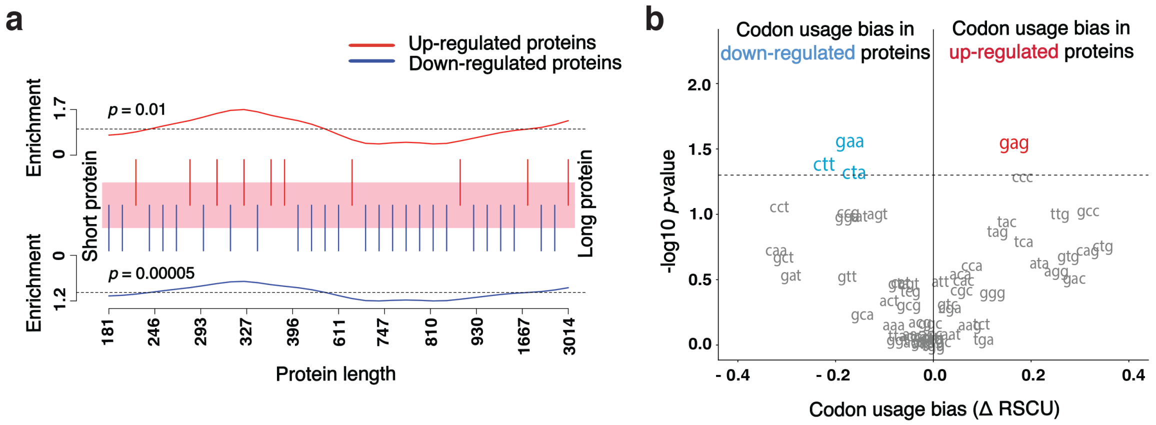

Supplementary Figure 14. Protein length and codon usage in developing brains of *Elp2H206R* vs. control mice. (**a**) Length distribution among up- and down-regulated proteins in the cortical extracts of *Elp2H206R* vs. wild-type 14.5 days post-coitum embryos. (**b**) Codon usage bias (AA-ending vs. AG-ending codons) in down- and up-regulated proteins. n = 3 animals per genotype. Statistical analysis: Wilcoxon test. Statistically significant differences are indicated in the image. Source data are provided as a Source Data file.

Supplementary Table 1. Main clinical features of patients carrying disease-causing *ELP2* variants

	Patient 1 (Cohen et al, 2015)	Patient 2 (Cohen et al, 2015)	Patient 3	Patient 4	Patient 5	Patient 6	Patient 7	Patient 8
***ELP2* variants**	p. H206R + p. R462W	p. H206R + p. R462W	p. L98Ffs*10 + p. T405I	p. L98Ffs*10 + p. T405I	p. H206R + p. N506Kfs*8	p. R462Q + p. Q553*	p. L444S + p. R820Sfs*3	p. R462Q (homozygous)
**Sex, age**	Male, 30 y	Male, deceased: 25 y (accidental choking)	Male, 18 m	Female, deceased: 4,5 m (respiratory insufficiency)	Male, 29 m	Male, 17 y, 9 m	Female, 25 y	Male, 17 y, 3 m
**Prenatal/neonatal course**	Hypotonia	Uncomplicated	Hypotonia, asphyxia, breathing difficulty, progressing heart insufficiency	Hypotonia, asphyxia, breathing difficulty, progressing heart insufficiency, oedema	Hypotonia	Uncomplicated	Uncomplicated	Uncomplicated
**Developmental delay**	Severe - profound	Severe - profound	Severe - profound	Unable to evaluate	Severe	Severe - profound	Unable to evaluate (most likely severe)	Severe - profound
**Intellectual disability**	Severe	Severe	NA	NA	NA	Profound	Severe	Severe
**Age of sitting/ walking**	14 m/9 y, later lost the ability to walk	15 m/never achieved	Never achieved	Never achieved	18 m/not yet walking	Never achieved	Unknown	10 m/never achieved
**Language abilities**	~ 50 words, lost meaningful spoken language	Non-verbal	Non-verbal	Non-verbal	Non-verbal	Non-verbal	4-5 y	Non-verbal
**Use of hands**	Purposeful hand use	Purposeful hand use	No targeted movements, poor grasping function	Uncoordinated movements	Purposeful hand use, clumsy, poor pincer grasp, stereotypies	Stereotypies: hand rubbing and wringing	Purposeful hand use	Stereotypies: hand wringing and flapping
**Behaviour/ASD**	Severe self-injury/ ASD	Severe self-injury/ ASD	Excessive sleepiness	Excessive sleepiness	ASD	Shaking/laughing episodes	Aggressivity/ASD	Self-stimulatory behabiour, hyperactive/ASD
**Feeding difficulty**	No	No	Yes, PEG-tube since 10 m, reflux	Yes, PEG-tube since 5m	Yes, PEG-tube since 23 m	Yes, PEG-tube	No	Yes
**Neurological exam**	Truncal hypotonia, spastic diplegia, choreoathetosis, ataxia	Truncal hypotonia, spastic diplegia, choreoathetosis	Truncal hypotonia, spastic paraplegia	Dystonia	Truncal hypotonia, spastic diplegia	Spastic tetraplegia	Normal	Hypotonia of extremities, paroxysmal generalized dystonia
**Ophtamological features**	Normal vision	Normal vision	CVI, unilateral coloboma	Poor eye contact, bilateral colobomas	CVI, foveal hypoplasia, nystagmus, dysconjugate gaze, exophoria	Normal vision, nystagmus	Normal vision	CVI, nystagmus
**Epilepsy**	No	No	Yes	Suspected, not confirmed	No	Yes	Yes	Yes
**Age of onset/ seizure type**	NA	NA	9 m/IS, SE	NA	NA	17 y, 9m/GTCS, SE	14 y/GTCS	4 y, 9m/nocturnal GTCS
**EEG**	Normal	Normal	Modified hypsarrhythmia	Normal	Mild diffuse slowing	Normal	Waves of generalized polyspikes	Moderate diffuse slowing
**AED received**	NR	NR	VGB, LEV, ZNS, HD	NR	NR	LEV	VPA, ZNS, LEV, PB	NR
**Seizure frequency**	NA	NA	Weekly	NA	NA	Unknown	Weekly	Monthly
**Brain MRI or UL**	Normal	Normal	UL: general atrophy, bilateral cysts in surrounding lateral ventricles	MRI: general atrophy, subependymale pseudocysts	UL: choroid plexus cyst - disappeared before birth MRI: delayed but improving myelination, thin corpus callosum	MRI: profound cerebellar, brainstem and supratentorial cortical atrophy, WM volume loss with secondary ventriculomegaly and increased extra-axial CSF spaces, microcephaly	Normal	MRI: simplified gyral pattern, cortical atrophy, white matter atrophy
**Congenital malformations/ additional features**	None	None	VSD, ASD*, PDA/ renal hypoplasia	VSD, ASD*, PDA/ broncho- and tracheomalacia, rectal prolapse, umbilical hernia	None/stridor, larygomalacia from 1 m, sleep apnea	None	None	None

Abbreviations: AED – antiepileptic drugs, ASD - autism spectrum disorder, ASD* - atrial septal defect, CVI - cortical visual impairment, DD - developmental delay, EEG – electroencephalography, GTCS - generalized tonic-clonic seizure, HD - hydrocortisone, ID - intellectual disability; IS - infantile spasms, LEV - levetiracetam, m - months, MRI - magnetic resonance imaging, NA – not applicable, NR – not required, PB - phenobarbital, PDA - patent ductus arteriosus, PEG - percutaneous endoscopic gastrostomy, SE - status epilepticus, UL - ultrasound, VGB - vigabatrin, VPA - valproic acid, VSD - ventricular septal defect, y - years, ZNS – zonisamide.

Supplementary Table 2. Volume changes in the *Elp2H206R* mouse brain

	Relative change in the volume of mouse brain regions (% of the total brain volume; mean ± SEM)		
**Structure**	**wild-type**	***Elp2H206R***	**Adjusted p-value**
Hippocampus	5.867633 ± 0.065787	6.354618 ± 0.111298	0.050803
External capsule	3.093557 ± 0.102202	2.672847 ± 0.145684	0.308714
Caudate putamen	5.933934 ± 0.061846	6.024996 ± 0.073479	0.846351
Anterior Commisure	0.201652 ± 0.002974	0.2597788 ± 0.014108	0.017915
Globus Pallidus	0.543824 ± 0.028228	0.4389170 ± 0.018203	0.223214
Internal Capsule	0.422742 ± 0.021723	0.2353112 ± 0.010209	0.002993
Thalamus	6.399709 ± 0.093350	5.894091 ± 0.081598	0.061725
Cerebellum	11.941060 ± 0.181416	10.61362 ± 0.314935	0.054305
Superior Colliculi	2.080718 ± 0.058053	2.119778 ± 0.093725	0.609423
Ventricles	0.247034 ± 0.008119	0.3528514 ± 0.02239	0.950982
Hypothalamus	3.082076 ± 0.065848	4.383420 ± 0.172950	0.000761
Inferior Colliculi	1.337301 ± 0.054331	1.455590 ± 0.060843	0.723786
Central gray	1.164943 ± 0.029093	1.248244 ± 0.085077	0.840945
Neocortex	33.282940 ± 0.303790	31.33459 ± 0.230353	0.025144
Amygdala	2.362849 ± 0.058855	2.705513 ± 0.113525	0.184297
Olfactory Bulb	5.578876 ± 0.178943	5.677643 ± 0.497516	0.950982
Brain stem	10.247510 ± 0.186211	10.89033 ± 0.35135	0.571517
Rest of Midbrain	2.760917 ± 0.039672	2.891464 ± 0.056690	0.515425
Basal Forebrain Septum	2.918191 ± 0.071703	3.926801 ± 0.132316	0.001559
Fimbria	0.532533 ± 0.015699	0.5195933 ± 0.021863	0.950982

Sample registration to the BNL C57BL/6J adult mouse brain atlas enabled model-based segmentation and volumetric measurement of listed brain structures in *Elp2H206R* homozygous mice and their wild-type littermates ($n = 6$) at postnatal day 21-23. Statistically significant differences are shown in red. Statistical analysis: two-tailed t-test with Holm-Sidak correction.

Supplementary Table 3. Increased connectivity in *Elp2H206R* mouse brain

Node	Node degree	Node	Node degree
Cerebellum L	11	Subiculum R	2
Ectorhinal cortex R	9	Secondary motor cortex R	2
Secondary visual cortex lateral L	8	Ventromedial orbital cortex L	2
Caudate putamen L	8	Secondary somatosensory cortex R	2
Anterior commissure	8	Secondary visual cortex mediolateral R	2
Dorsal fornix	8	Insular cortex R	2
Primary somatosensory cortex R	7	Perirhinal cortex L	2
Septum L	7	Claustrum L	2
Forceps minor of the cospus callosum L	7	Endopiriform nucleus L	2
Dorsal hippocampal commisure inclusion	7	Piriform nucleus L	2
Primary visual cortex L	6	Amygdala L	2
Primary visual cortex R	6	Amygdala R	2
Perirhinal cortex R	6	Hippocampus L	2
Clustrum R	6	Cingulum R	2
Caudate putamen R	6	Dorsal fornix L	2
Septum R	6	Lateral olfactory tract L	2
Primary motor cortex R	5	Pyramidal tract L	2
Secondary somatosensory cortex L	5	Pyramidal tract R	2
Temporal association area R	5	Fornix	2
Secondary visual cortex lateral R	5	Dorsolateral orbital cortex R	1
Insular cortex L	5	Frontal cortex, area 3 R	1
Ectorhinal cortex L	5	Frontal association cortex L	1
Thalamus L	5	Lateral orbital cortex R	1
Inferior colliculus L	5	Primary motor cortex L	1
Cerebellum R	5	Secondary motor cortex L	1
Dorsal hippocampal fissure L	5	Endopiriform nucleus R	1
Dorsal hippocampal fissure R	5	Lateral globus pallidus L	1
Corpus callosum (inside midline)	5	Hypothalamus L	1
Corpus callosum/external capsule R	5	Hypothalamus R	1
Entorhinal cortex R	4	Superior colliculus R	1
Temporal association area L	4	Dorsal fornix R	1
Retrosplenial area R	4	Lateral olfactory tract R	1
Hippocampus R	4	Internal capsule L	1
Inferior colliculus L	4	Hindbrain L	1
Internal capsule R	4		
Subiculum L	3		
Entorhinal cortex L	3		
Frontal association cortex R	3		
Primary somatosensory cortex L	3		
Secondary auditory cortex R	3		
ANterior cingulate L	3		
Retrosplenial area L	3		
Piriform nucleus R	3		
Accumbens nucleus L	3		
Thalamus R	3		
Cingulum L	3		
Forceps minor of the corpus callosum R	3		
Ventral hippocampal commisure inclusion	3		
Corpus callosum/external capsule L	3		

Structural connectome generated via whole brain white matter tractography of wild-type and *Elp2H206R* homozygous mice (n = 6 per genotype) at postnatal day 21-23. Increased connectivity in listed brain structures (nodes) in *Elp2H206R* mice is relative to their wild-type littermates. Node degree represents the number of abnormal connections associated with the node. Abbreviations: L - left, R - right.

Supplementary Table 4. Decreased connectivity in *Elp2H206R* mouse brain

Node	Node degree
Caudate putamen R	5
Dorsal fornix L	5
Dorsal fornix R	4
Internal capsule R	4
Dorsal hippocampal commisure inclusion	4
Dorsal fornix	4
Corpus callosum (inside midline)	4
Lateral globus pallidus R	3
Thalamus R	3
Superior colliculus R	3
Corpus callosum/external capsule R	3
Hippocampus R	2
Cingulum R	2
Dorsal hippocampal fissure L	2
Corpus callosum/external capsule L	2
Frontal association cortex R	1
Ventromedial orbital cortex R	1
Periaqueductal grey R	1
Dorsal hippocampal fissure R	1
Pyramidal tract R	1

Structural connectome generated via whole brain white matter tractography of wild-type and *Elp2H206R* homozygous mice (n = 6 per genotype) at postnatal day 21-23. Decreased connectivity in listed brain structures (nodes) in *Elp2H206R* mice is relative to their wild-type littermates. Node degree represents the number of abnormal connections associated with the node. Abbreviations: L - left, R - right.

Supplementary Table 5. Sequences of primers used in this study

Primer	Sequence 5'- 3'
Elp2H206R_for and Elp2KO_for (genotyping)	CGAGTGCCTTACAGCATACCCGC
Elp2H206R_for and Elp2KO_rev (genotyping)	AACCTGCTTCTCACCCCACAGTGC
Elp2R464W_for (genotyping)	TTCAGGGCCTTCCTTCCACTTGA
Elp2R464W_rev (genotyping)	TGCTGTTTTAGAGTCTTGAACTCATTTG
hElp2_L98F_for	GAAATCGAGGATAACCAGCTGTTCAAAGCTGTTCACCTGCAGG
hElp2_L98F_rev	CCTGCAGGTGAACAGCTTTGAACAGCTGGTTATCCTCGATTTC
hElp2_H206R_for	GTTCTGAGCCTGTGCGGTCGCGAAGATTGGATCCGTG
hElp2_H206R_rev	CACGGATCCAATCTTCGCGACCGCACAGGCTCAGAAC
hElp2_T405I_for	GGAAGGCGAGTTCATCATCATCGTTGGCACCGACCAGACC
hElp2_T405I_rev	GGTCTGGTCGGTGCCAACGATGATGATGAACTCGCCTTCC
hElp2_R464W_for	CTGATGAAAAAGTTCTGTGGGTGTTCAGCGCGCCG
hElp2_R464W_rev	CGGCGCGCTGAACACCCACAGAACTTTTTCATCAG
hElp2_R464Q_for	CTGATGAAAAAGTTCTGTCAATGTTCAGCGCGCCG
hElp2_R464Q_rev	CGGCGCGCTGAACACTTGCAGAACTTTTTCATCAG
mElp2_L98F_for	GAACTAGAGAATAATCAGGTTTTTAAATCAGTCCGTCTCCAAGGCC
mElp2_L98F_rev	GGCCTTGGAGACGGACTGATTTAAAAACCTGATTATTCTCTAGTTC
mElp2_H206R_for	CTTTCTCTCTGCGGACGCGAGGACTGGATAAGAG
mElp2_H206R_rev	CTCTTATCCAGTCCTCGCGTCCGCAGAGAGAAAG
mElp2_T404I_for	CAGAGGGAGAGTTCATCATCATTACCAGCACCGATCAGACCAC
mElp2_T404I_rev	GTGGTCTGATCGGTGCTGGTAATGATGATGAACTCTCCCTCTG
mElp2_R464W_for	GCAGACGAGAAAGTTCTTTGGGTTTTTTCTGCTCCTCGG
mElp2_R464W_rev	CCGAGGAGCAGAAAAAACCCAAAGAACTTTCTCGTCTGC
mElp2_R464Q_for	GCAGACGAGAAAGTTCTTCAAGTTTTTTCTGCTCCTCGG
mElp2_R464Q_rev	CCGAGGAGCAGAAAAAACTTGAAGAACTTTCTCGTCTGC

Abbreviations: for – forward, rev – reverse.
